# Prevalence of the CRISPR-cas system and its association with antibiotic resistance in clinical *Klebsiella pneumoniae* isolates

**DOI:** 10.1186/s12879-024-09451-5

**Published:** 2024-06-03

**Authors:** Hiva Kadkhoda, Pourya Gholizadeh, Reza Ghotaslou, Tahereh Pirzadeh, Mohammad Ahangarzadeh Rezaee, Edris Nabizadeh, Hadi Feizi, Hossein Samadi Kafil, Mohammad Aghazadeh

**Affiliations:** 1 Student Research Committee, Tabriz University of Medical Sciences, Tabriz, Iran; 2 Department of Medical Microbiology, Faculty of Medicine, Tabriz University of Medical Sciences, Tabriz, Iran; 3 Digestive Disease Research Center, Ardabil University of Medical Sciences, Ardabil, Iran; 4 Zoonoses Research Center, Ardabil University of Medical Sciences, Ardabil, Iran; 5 Infectious and Tropical Diseases Research Center, Tabriz University of Medical Sciences, Tabriz, Iran; 6 Department of Medical Microbiology, Aalinasab Hospital, Social Security Organization, Tabriz, Iran; 7 Drug Applied Research Center, Tabriz University of Medical Sciences, Tabriz, Iran; 8 Immunology Research Center, Tabriz University of Medical Sciences, Tabriz, Iran

**Keywords:** CRISPR-Cas system, *Klebsiella pneumoniae*, Extended-spectrum β-lactamases

## Abstract

**Background and objective(s):**

CRISPR-Cas is a prokaryotic adaptive immune system that protects bacteria and archaea against mobile genetic elements (MGEs) such as bacteriophages plasmids, and transposons. In this study, we aimed to assess the prevalence of the CRISPR-Cas systems and their association with antibiotic resistance in one of the most challenging bacterial pathogens, *Klebsiella pneumoniae*.

**Materials and methods:**

A total of 105 *K. pneumoniae* isolates were collected from various clinical infections. Extended-spectrum β-lactamases (ESBLs) phenotypically were detected and the presence of ESBL, aminoglycoside-modifying enzymes (AME), and CRISPR-Cas system subtype genes were identified using PCR. Moreover, the diversity of the isolates was determined by enterobacterial repetitive intergenic consensus (ERIC)-PCR.

**Results:**

Phenotypically, 41.9% (44/105) of the isolates were found to be ESBL producers. A significant inverse correlation existed between the subtype I-E CRISPR-Cas system’s presence and ESBL production in *K. pneumoniae* isolates. Additionally, the frequency of the ESBL genes *bla*_*CTX−M1*_ (3%), *bla*_*CTX−M9*_ (12.1%), *bla*_*SHV*_ (51.5%), and *bla*_*TEM*_ (33.3%), as well as some AME genes such as *aac(3)-Iva* (21.2%) and *ant(2’’)-Ia* (3%) was significantly lower in the isolates with the subtype I-E CRISPR-Cas system in comparison to CRISPR-negative isolates. There was a significant inverse correlation between the presence of ESBL and some AME genes with subtype I-E CRISPR-Cas system.

**Conclusion:**

The presence of the subtype I-E CRISPR-Cas system was correlated with the antibiotic-resistant gene (ARGs). The isolates with subtype I-E CRISPR-Cas system had a lower frequency of ESBL genes and some AME genes than CRISPR-negative isolates.

## Introduction

Acquisition of DNA elements such as fitness, antibiotic resistance, and virulence genes through horizontal gene transfer (HGT) is a crucial step in bacterial adaptation to various hosts and environments [1, 2]. Furthermore, many species of bacteria have developed an adaptive immune system known as clustered regularly interspaced short palindromic repeats and their associated Cas proteins (CRISPR-Cas), which helps to restrict the acquisition of external genetic elements and protect against invasive plasmids and bacteriophages [3, 4]. These defense systems are composed of a leader sequence, *cas* genes, and a CRISPR array. A CRISPR array typically consists of highly conserved short direct repeats (DR), separated by unique sequences (spacers) acquired from mobile genetic elements (MGEs) [5–7]. The number of *cas* genes in the CRISPR-Cas locus is variable and are often located next to CRISPR repeat-spacer arrays [8]. Based on the *cas* operon architecture, *cas* gene content, and Cas protein sequences, CRISPR-Cas systems are classified into two main classes, comprising 6 major types and 33 distinct subtypes [9]. The Cas proteins possess a variety of enzymatic domains with helicase, polymerase, or nuclease activity and are essential for the functioning of the CRISPR-Cas system [10, 11].

Acquiring new DNA, often encoded on MGEs like transposons and plasmids, is the mechanism by which many bacteria develop resistance to antibiotics. Investigations conducted on various bacteria have demonstrated the significance of CRISPR-Cas systems in the exchange of genetic material and their potential to impact the rate of evolution [12]. Palmer et al. [13] demonstrated a significant negative correlation between antibiotic resistance acquisition and the CRISPR-Cas system’s presence in *Enterococcus faecalis* isolates. Moreover, a few studies suggested that the CRISPR-Cas system could regulate the pathogenicity of bacteria. The CRISPR-Cas system in *Pseudomonas aeruginosa* allows for the modulation of biofilm formation, which is a crucial factor in the pathogenicity of a variety of microorganisms [14]. CRISPR-Cas system modulates the prophage’s contents in *Streptococcus pyogenes* and, consequently, its virulence [15]. However, Touchon et al. [16] demonstrated that the CRISPR-Cas system is not an effective barrier against antibiotic resistance and plasmid spreading in *Escherichia coli*. For these reasons, there is much potential for research into how this system affects various bacterial pathogens’ virulence and antibiotic resistance.

Owing to the extensive dissemination and high rate of antibiotic resistance, *Klebsiella pneumoniae* has become a predominant opportunistic pathogen in hospital environments [17]. High molecular weight plasmids are associated with hypervirulent phenotype and multidrug resistance in *K. pneumoniae* [18, 19]. So far, two types of CRISPR-Cas systems including type I (types I-F, I-E, and I-E*) and IV (primarily type IV-A) systems have been identified in *Klebsiella* spp. The type I CRISPR system is located mainly in chromosome, while the type IV system is exclusively found in plasmids [8, 20, 21]. Type I-E is the canonical type I-E CRISPR-Cas system (located in the *cysH-iap* region), containing a *cas* operon, consistent direct repeats (29 bp), and a CRISPR array (designated as CRISPR1) which is situated downstream of the *cas* genes. Type I-E* is variable in comparison to type I-E, which is located in the ABC transport system-glyoxalase area and occasionally has a transposase-encoding gene integrated into the *cas* operon. Besides, this type contains two CRISPR arrays (designated as CRISPR2 and CRISPR3, respectively) that bracket the *cas* genes [22–24]. The marker gene of the type I CRISPR-Cas system is *cas3*, and *cas1* is a universal *cas* gene in all CRISPR-Cas types [25]. Whether the CRISPR-Cas system in *K. pneumoniae* facilitates HGT or functions as an immune system is still a question. Therefore, this study aimed to determine the correlation between the presence of the CRISPR-Cas system and ESBL and aminoglycoside genes in clinical *K. pneumoniae* isolates.

## Materials and methods

### Isolation and identification of K. pneumoniae

In this prospective study, one hundred and five non-duplicate and non-consecutive clinical *K. pneumoniae* isolates were gathered from various clinical specimens including urine, sputum, blood, and wound from the patients admitted to Imam Reza Teaching and Treatment Hospital in Tabriz, Iran. These isolates were initially identified by conventional bacteriology tests such as gram staining, colony morphology, the reaction in triple sugar iron agar (TSA), lysine iron agar (LIA), citrate utilization, indole production, and motility. Subsequently, molecular identification was carried out using polymerase chain reaction (PCR) as described elsewhere [26]. Ultimately, the identified isolates were stored in Tryptic Soy Broth (TSB, Merk) containing 10% (v/v) glycerol and kept at -70 °C until used.

### Phenotypic detection of extended-spectrum β-lactamases (ESBLs) production

ESBL phenotypic detection was accomplished by the combination disk diffusion test (CDDT) in accordance with the Clinical and Laboratory Standards Institute (CLSI) guidelines [27]. Ceftazidime (30 µg), cefotaxime (30 µg), ceftazidime-clavulanic acid (30/10 µg), and ceftazidime-clavulanic acid (30/10 µg) disks (Mast Group Ltd., Merseyside, UK) were used in the CDDT. The discs were placed onto a Mueller Hinton agar (MHA) plate that had been inoculated with the test strain. When the diameter of the inhibition zone surrounding the combination disks was greater than that of the cefotaxime (30 µg) or ceftazidime (30 µg) disks alone by at least 5 mm, the isolates of *K. pneumoniae* were considered as ESBL-producers. *K. pneumoniae* ATCC 700,603 and *Escherichia coli* ATCC 25,922 were used as the positive and negative controls for the production of ESBLs, respectively.

### Genotypic detection of CRISPR and antibiotic resistance genes

The tissue buffer boiling method (0.05 M NaOH and 0.25% sodium dodecyl sulfate (SDS) was utilized to extract the total DNA of the isolates. The *CRISPR1*, *CRISPR2*, and *CRISPR3* genes were detected via PCR in order to confirm the existence of the CRISPR-Cas system. The *cas1* and *cas3* genes were also identified to detect the type of CRISPR-Cas system. Moreover, the presence of aminoglycoside-modifying enzymes (AME) and ESBL genes were identified using PCR amplification. Table 1 displays the primer sequences along with the amplified size. A 25 µl reaction mixture was used for the PCR amplification, which included 12.5 µL of Taq DNA Polymerase 2X Master Mix RED (Amplicon Co., Denmark), 1 µL of extracted DNA as the template, 1 µL of each primer (10 pmol), and 9.5 µL of DW in the BIO-RAD C1000 thermal cycler (Applied Biosystems, USA). A standard UV transilluminator was used to view the stained gels after the PCR products were electrophoresed on a 1.5% agarose gel in 1X TBE buffer.

**Table 1:** Oligonucleotide primer sequences used in the Study

Primer	Sequence (5′–3′)	Amplicon size (bp)		Reference
*16 S rRNA*	F: ATTTGAAGAGGTTGCAAACGAT R: TTCTGAAGTTTTCTTGTGTTC		130	[26]
*CRISPR1*	F: CGGTTCTTCGGGCTTAAACG R: CTGCTGCAATGACGCCAG		391	[65]
*CRISPR2*	F: TGTTCGCCGCTGAGTTTATG R: TACCACGCCAGTTACTACGC		459	[65]
*CRISPR3*	F: GACGCTGGTGCGATTCTTGAG R: CGCAGTATTCCTCAACCGCCT		1598	[65]
*cas1*	F: CTTTTGGCACGACGGAATCA R: TGGCGCTGGATGATGATTTG		381	[65]
*cas3*	F: GTCCCGACTAAAATGCGTCC R: CGTTGATGGCGGTGATGAAT		598	[65]
*blaTEM*	F: TGCGGTATTATCCCGTGTTG R: TCGTCGTTTGGTATGGCTTC		296	[62]
*blaSHV*	F: AGCCGCTTGAGCAAATTAAAC R: ATCCCGCAGATAAATCACCAC		713	[62]
*blaPER*	F: TGGGCTTAGGGCAGAAAG R: GAATACCTGGGCTCCGATAA		607	[66]
*CTX-M1*	F: CTCACGCTGTTGTTAGGAA R: ACGGCTTTCTGCCTTAGGTT		780	[67]
*CTX-M9*	F: ATGGTGACAAAGAGAGTGCA R: CCCTTCGGCGATGATTCTC		863	[68]
*ant(2˝)-Ia*	F: ATCTGCCGCTCTGGAT R: CGAGCCTGTAGGACT		404	[69]
*aac(3´)-IIa*	F: ATGCATACGCGGAAGGC R: TGCTGGCACGATCGGAG		822	[69]
*aac(3´)-IVa*	F: GTGTGCTGCTGGTCCACAGC R: AGTTGACCCAGGGCTGTCGC		627	[70]
*aac(6)´-Ib*	F: ATGACTGAGCATGACCTTG R: AAGGGTTAGGCAACACTG		524	[69]
*aph(3´)-Ia*	F: CGAGCATCAAATGAAACTGC R: GCGTTGCCAATGATGTTACAG		623	[71]
*aac(3´)-Ia*	F: GACATAAGCCTGTTCGGTT R: CTCCGAACTCACGACCGA		372	[71]
*ant(4’)-IIa*	F: ATCGTCTGCGAGAAGCGTAT R: TAAAACGCCTATCCGTCACC		839	[72]

### Analysis of genotype by enterobacterial repetitive intergenic consensus (ERIC)-PCR

The ERIC-PCR method was used to assess the isolates’ genetic relatedness to one another. The single primer ERIC1 with a sequence of 5’-ATGTAAGCTCCTGGGGATTCAC-3’ was used for genotype of all isolates. ERIC-PCR was performed in a volume of 25 µl containing 12.5 µL of TEMPase DNA Polymerase Hot Start 2x Master Mix BLUE PCR (Amplicon Co., Denmark), 2 µL of template DNA, 1 µL of each primer (10 pmol), and 9.5 µL of nuclease-free water. The thermal cycling conditions were as follows: initial denaturation at 94ºC for 5 min, followed by 35 repeated cycles of DNA denaturation at 94ºC for 30 s, annealing at 48ºC for 1 min, extension of primer at 72 ºC for 2 min, and a final extension at 72 °C for 5 min. Following electrophoresis on 1.5% (w/v) agarose gel, the amplified fragments were stained with DNA-safe stain (Sinaclon Co., Tehran, Iran), visualized with UV light, and captured with an ultraviolet gel documentation device (Uvitec, UK). New England Biolabs’ 100 bp DNA ladders were used as molecular size markers to estimate product size. The similarity between strains was found based on the analysis of the banding by GelJ software, and dendrograms were generated using the Dice similarity method and the Unweighted Pair Group Method with Arithmetic Averages (UPGMA) technique.

### Statistical analyses

Descriptive statistics were analyzed by SPSS software (version 27.0 SPSS Inc., Chicago, IL, USA). Pearson chi-square or One-tailed Fisher’s exact tests (when one or more of the cell counts is less than 5) were used to compare the occurence of the CRISPR-Cas system and its subtypes and the presence of ESBL and aminoglycoside genes among *K. pneumoniaee* isolates. In addition, the correlation between the presence of CRISPR-Cas systems and different ARGs was calculated by Spearman’s rank correlation coefficient among the isolates. The discriminatory power was measured by http://insilico.ehu.eus/. The p-value < 0.05 was considered statistically significant.

## Results

### Bacterial isolates

From 2022 to 2023, 105 clinical *K. pneumoniae* isolates collected were initially identified by conventional biochemical tests. All isolates had a positive for the *16 S rRNA* gene and were confirmed at the molecular level as *K. pneumoniae*. These isolates were recovered from diverse clinical specimens including urine (*n* = 49, 46.6%), sputum (*n* = 27, 25.7%), blood (*n* = 19, 18.1%), and wound (*n* = 10, 9.5%). The isolates were obtained from 56 (53.3%) males and 49 (46.7%) females, aged 4 to 84 years, with a mean of 57.6 ± 22.6 years. The hospital source of the isolates encompassed: internal (49.5%) followed by intensive care unit (ICU) (21.9%), surgery (12.4%), burn (10.5%), and infection (5.7%) wards.

### Distribution of CRISPR-Cas system in K. pneumoniae

PCR was utilized for the detection of *CRISPR1*, *CRISPR2*, *CRISPR3, cas1*, and *cas3* genes. A three-group division of all isolates was made according to the distribution of CRISPR-Cas systems: (1) isolates carrying subtype I-E* CRISPR-Cas system; (2) isolates carrying subtype I-E CRISPR-Cas system; and (3) isolates lacking the CRISPR-Cas system. PCR analysis of the CRISPR-Cas system’s subtype genes revealed that 36 (34.2%) out of 105 isolates contained the CRISPR-Cas system. Out of them, 33 (31.4%) isolates possessed the subtype I-E CRISPR-Cas system, and 3 (2.8%) isolates possessed the subtype I-E* system. Type I-E and subtype I-E* did not co-exist in any of the examined isolates. All of the *Cas3*-positive isolates, we also found, had at least one CRISPR array (*CRISPR1*, *CRISPR2* or *CRISPR3*) and were devoid of *Cas1*.

### Correlation between CRISPR-Cas system and ESBL production

Of the 105 *K. pneumoniae* isolates that were not susceptible to cefotaxime, 44 (41.9%) were found to be ESBL producers using the combination disk diffusion test (CDDT). The majority of isolates containing the CRISPR system were unable to produce ESBLs. Of the isolates containing subtype I-E CRISPR-Cas system, only 5 (15.2%) were ESBL producers, whereas the remaining 28 (84.8%) isolates did not. Statistical analysis revealed a significant inverse correlation between the subtype I-E CRISPR-Cas system’s presence and ESBL production in *K. pneumoniae* isolates (P-value > 0.001, correlation coefficient = -0.367).

### Genetic context of ESBL and CRISPR-Cas systems

PCR results showed that 88.5% (93/105) of the isolates contained ESBL genes. The *bla*_*SHV*_ gene (73.3%, 77/105) was the predominant ESBL gene in the *K. pneumoniae* isolates, followed by *bla*_*TEM*_ (64.8%, 68/105), *bla*_*CTX−M9*_ (31.4%, 33/105) and *bla*_*CTX−M1*_ (14.3%, 15/105). The *bla*_*PER*_ gene was not found in any of the tested isolates. In addition, there were 11 different patterns associated with the distribution of ESBL genes (Table 2). At least two or more ESBL genes were present in most isolates containing ESBL genes. The combination of *bla*_*CTX−M9*_, *bla*_*TEM*_, and *bla*_*SHV*_ was the most common (26.9%, 25/93), followed by *bla*_*TEM*_ and *bla*_*SHV*_ (21.5%, 20/93). 29 (31.2%) out of 93 isolates also had a single ESBL gene; 15 of them harbored *bla*_*SHV*_, 12 harbored *bla*_*TEM*_, and 2 harbored *bla*_*CTX−M1*_. Furthermore, there was a significant correlation between the presence of subtype I-E CRISPR-Cas system and ESBL genes (Table 3). The results demonstrated that the frequency of the ESBL genes *bla*_*CTX−M1*_ (1/33, 3.0%), *bla*_*CTX−M9*_ (4/33, 12.1%), *bla*_*SHV*_ (17/33, 51.5%), and *bla*_*TEM*_ (11/33, 33.3%) was significantly lower in the isolates containing the subtype I-E CRISPR-Cas system in comparison to CRISPR-negative isolates (*P* = 0.059, *P* = 0.011, *P* = 0.046, *P* < 0.001, respectively).

**Table 2:** Distribution of the genes associated with ESBL and AME in *K. pneumoniae* isolates

Type of Isolate	Type of Gene	Number of Isolates (%)
Isolates containing ESBL genes (*N* = 93)	*bla*_*TEM*_	12 (12.9%)
	*bla*_*SHV*_	15 (16.2%)
	*bla*_*CTX−M1*_	2 (2.1%)
	*bla*_*CTX−M9*_, *bla*_*TEM*_	1 (1.1%)
	*bla*_*CTX−M9*_, *bla*_*SHV*_	5 (5.4%)
	*bla*_*CTX−M1*_, *bla*_*SHV*_	3 (3.2%)
	*bla*_*CTX−M1*_, *bla*_*TEM*_	1 (1.1%)
	*bla*_*TEM*_, *bla*_*SHV*_	20 (21.5%)
	*bla*_*CTX−M1*_, *bla*_*TEM*_, *bla*_*SHV*_	7 (7.5%)
	*bla*_*CTX−M9*_, *bla*_*TEM*_, *bla*_*SHV*_	25 (26.9%)
	*bla*_*CTX−M9*_, *bla*_*CTX−M1*_, *bla*_*TEM*_, *bla*_*SHV*_	2 (2.1%)
Isolates containing AME genes (*N* = 91)	*aac(6´)-Ib*	10 (11%)
	*ant(2’’)-Ia*	1 (1.1%)
	*aac(3)-IVa*	2 (2.2%)
	*aac(6´)-Ib*, *aac(3)-IVa*	22 (24.2%)
	*aac(6´)-Ib*, *aac(3)-Ia*	18 (19.7%)
	*aac(6´)-Ib*, *aph(3´)-Ia*	13 (14.3%)
	*aac(6´)-Ib*, *ant(2’’)-Ia*	2 (2.2%)
	*aac(6´)-Ib*, *aac(3)-IVa*, *aac(3)-Ia*	7 (7.7%)
	*aac(6´)-Ib*, *aph(3´)-Ia*, *ant(2’’)-Ia*	2 (2.2%)
	*aac(6´)-Ib*, *aac(3)-IVa*, *aph(3´)-Ia*	1 (1.1%)
	*aac(6´)-Ib*, *aac(3)-IVa*, *ant(2’’)-Ia*	3 (3.3%)
	*aac(6´)-Ib*, *aac(3)-Ia*, *ant(2’’)-Ia*	5 (5.5%)
	*aac(6´)-Ib*, *aac(3)-Iva*, *aac(3)-Ia*, *ant(2’’)-Ia*	1 (1.1%)
	*aac(6´)-Ib*, *aac(3)-Iva*, *aph(3´)-Ia*, *ant(2’’)-Ia*	4 (4.4%)

**Table 3:** The association of drug-resistance genes with CRISPR-Cas Systems in *K. pneumoniae* isolates

Subtype I-E CRISPR-Cas system			Subtype I-E^∗^ CRISPR-Cas System			CRISPR-Cas negative	
Drug Resistance Genes	Present *n* = 33	Absent *n* = 72	p	Present *n* = 3	Absent *n* = 102	p	*n* = 69
*bla*_*CTX−M1*_	1	14	**0.04**	1	14	0.33	13
*bla*_*CTX−M9*_	4	29	**0.01**	1	32	0.68	27
*bla*_*TEM*_	11	57	**> 0.001**	2	66	0.71	55
*bla*_*SHV*_	17	60	**0.01**	2	75	0.97	58
*aac(6´)-Ib*	28	60	0.14	2	86	0.41	58
*aac(3)-Ia*	7	24	0.37	1	30	0.65	23
*aac(3)-IVa*	7	33	**0.05**	1	39	0.67	32
*aph(3´)-Ia*	8	12	0.20	0	20	0.52	12
*ant(2’’)-Ia*	1	17	**0.01**	2	16	0.08	15

Bold font indicates statistically significant correlations (*p* < 0.05)

### CRISPR-Cas systems and AME genes

**Fig. 1:**
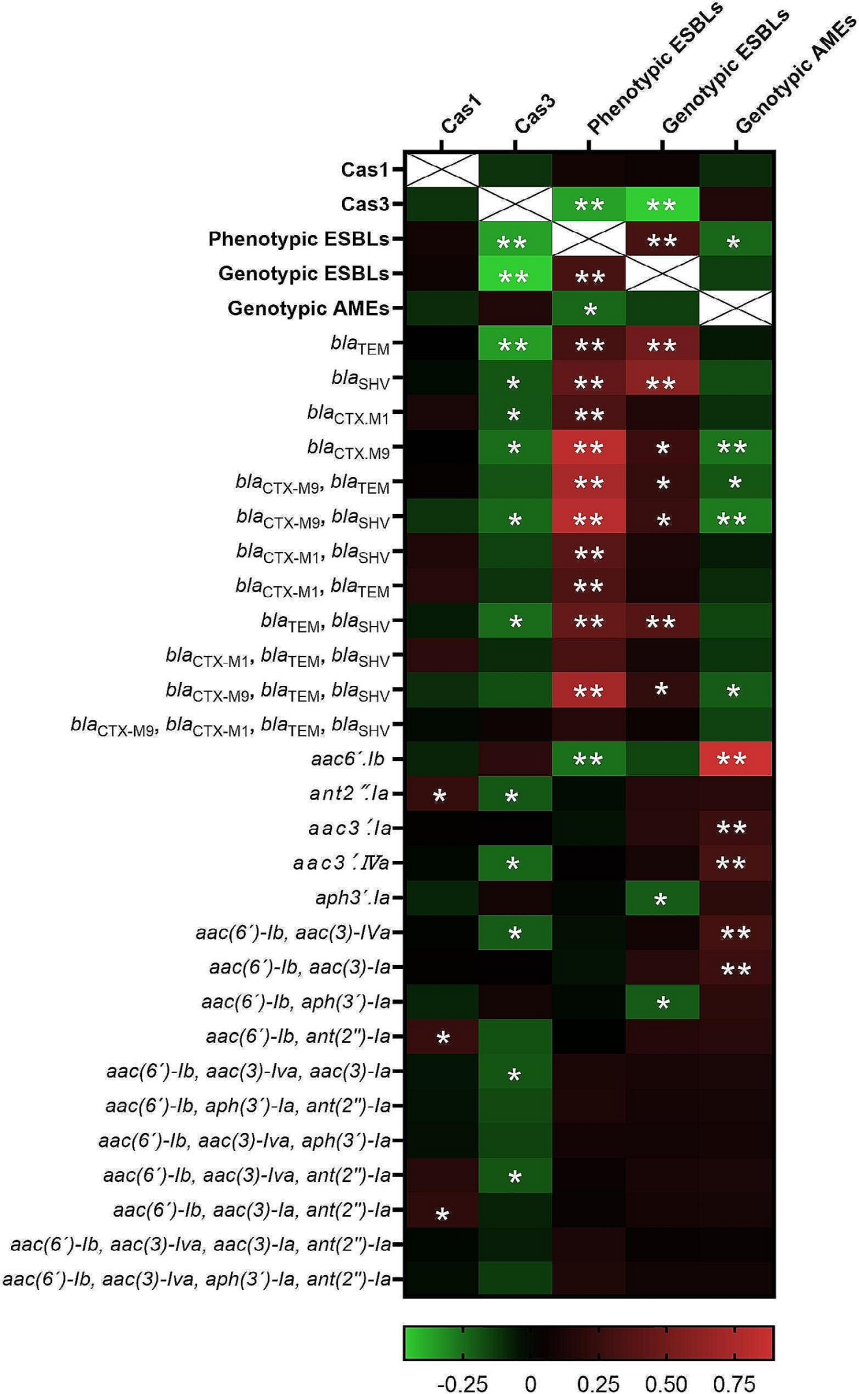
The correlations between CRISPR systems and phenotypic and genotypic ESBLs and AME genes among the *K. pneumoniae* isolates. The presence of most of the ESBL and aminoglycoside genes was correlated to the absence of the *cas3* gene among the isolates (P-value < 0.05). In addition, the phenotypic ESBLs were directly correlated to genotypic ESBLs (*P*-value < 0.001) and inversely correlated to genotypic AMEs (*P*-value < 0.05). Furthermore, genotypic AMEs were directly correlated to aminoglycoside resistance genes (*P*-value < 0.001). The green stains are demonstrated the inverse correlations and the red stains are demonstrated direct correlations. The correlations were tested using Spearman’s rank test. *: *P*-value < 0.05; **: *P*-value < 0.001

PCR results also revealed that 91 (88.6%) out of 105 isolates contained AME genes. The *aac(6´)-Ib* gene (83.8%, 88/105) was the most common AME gene, followed by *aac(3)-IVa* (38.1%, 40/105), *aac(3)-Ia* (29.5%, 31/105), *aph(3´)-Ia* (19%, 20/105) and *ant(2’’)-Ia* (17.1%, 18/105). *ant(4’)-IIa* and *aac(3)-IIa* genes weren’t detected in any isolates. In addition, there were 14 different patterns associated with the distribution of AME genes (Table 2). At least two or more AME genes were present in most isolates containing AME genes. The most common combination of AME genes was *aac(3)-IVa* and *aac(6´)-Ib* with 24.2% (22/91), followed by *aac(3)-Ia* and *aac(6´)-Ib* (19.7%, 18/91) and *aac(6´)-Ib* and *aph(3´)-Ia* (14.3%, 13/91). Also, 14.3% (13/91) of the isolates possessed only one of the examined AME genes, of which 10 harbored *aac(6´)-Ib*, 2 harbored *aac(3)-IVa*, and 1 harbored *ant(2’’)-Ia*. Moreover, there was a significant correlation between the presence of subtype I-E CRISPR-Cas system and some AME genes (Table 3). The results revealed that the frequency of the AME genes *aac(3)-Iva* (7/33, 21.2%) and *ant(2’’)-Ia* (1/33, 3%) was significantly lower in the isolates with the subtype I-E CRISPR-Cas system in comparison to CRISPR-negative isolates (*P* = 0.05 and *P* = 0.01, respectively). The association between the presence of CRISPR systems and ESBL production and ESBLs and AME genes among the *K. pneumoniae* isolates is shown in Fig. 1.

### ERIC-PCR analysis

**Fig. 2:**
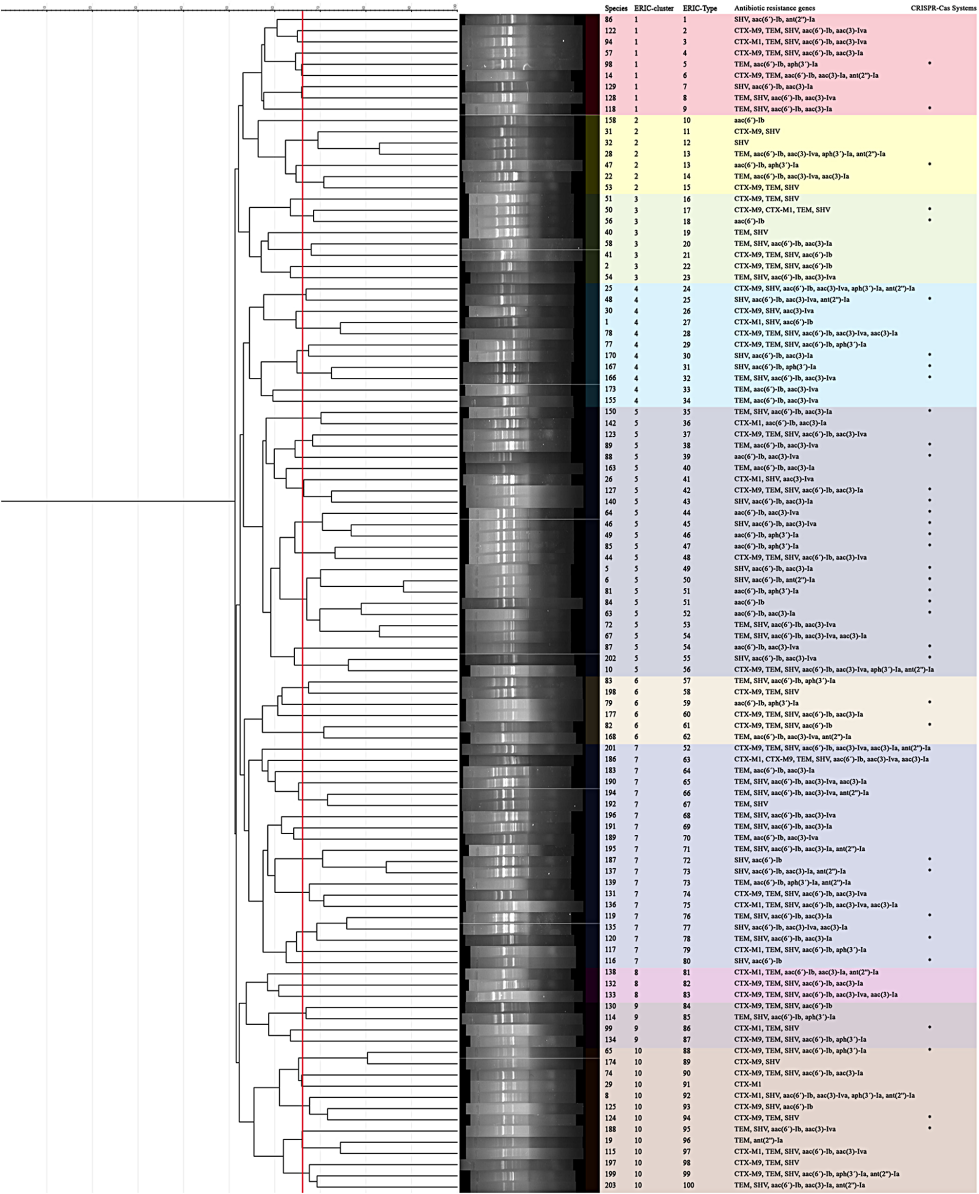
The genomic diversity of the clinical *K. pneumoniae* isolates by using ERIC-PCR at 80% similarity cut-off value

The determination of genomic diversity of 105 clinical *K. pneumoniae* isolates demonstrated that there were one hundred ERIC types, including 95 singletons and 5 common, using ERIC-PCR at an 80% similarity cut-off value with a discriminatory power of 0.9991 (Fig. 2). In addition, there were 10 clusters at 53% similarity cut-off value with a discriminatory power of 0.8703. Generally, the number of bands in the electronic analysis of the PCR products ranged from 4 to 13 with the sizes ranging from about 300 bp to more than 1500 bp. Out of 105 isolates, a total of 9 (8.6%) isolates belonged to cluster 1, 7 (6.7%) isolates to cluster 2, 8 (7.6%) to cluster 3, 11 (10.5%) to cluster 4, 24 (22.9%) to cluster 5, 6 (5.7%) to cluster 6, 20 (19%) to cluster 7, 3 (2.9%) to cluster 8, 4 (3.8%) to cluster 9 and 13 (12.4%) to cluster 10. The isolates were not significant among the ERIC clusters (*P* = 0.1285). There were no isolates with CRISPR-Cas systems in cluster 8 and cluster 5 had the most isolates containing the CRISPR-Cas system. In addition, the AME and ESBL genes distribution pattern demonstrated no significant association with ERIC clusters (*P* = 0.1312 and *P* = 0.738, respectively).

## Discussion

A recent study examined CRISPR-Cas systems in the genomes of *K. pneumoniae* [28]. According to bioinformatics studies, only 6 out of 52 *K. pneumoniae* strains with the available draft or complete genomes had a complete CRISPR-Cas system, indicating a limited distribution of the CRISPR-Cas systems in *K. pneumoniae.* Out of 116 spacer sequences, 38 spacers were found to be extremely similar to the bacterial, plasmid, or phage genome sequences using blast search analysis [28]. In addition, Liao et al. [29] revealed that only 14.9% (25/168) of clinical *K. pneumoniae* isolates had the CRISPR-Cas system. There could be multiple reasons for the restricted distribution of the CRISPR-Cas systems in *K. pneumoniae*. First, the loss of the CRISPR-Cas system, may be due to strong selective pressure to acquire antibiotic resistance or virulence genes [30]. Secondly, the CRISPR-Cas system itself might be an MGE associated with various habitats, which could be transferred into *K. pneumoniae* strains [31]. Thirdly, CRISPR-Cas systems distribution is non-random and Multilocus sequence typing (MLST)-dependent [29, 32]. As the CRISPR-Cas system appears not to be widely prevalent in *K. pneumoniae* species, it remains to be determined how the system’s presence or absence could contribute to the evolution of *K. pneumoniae* strains [33].

Based on Cas1 and Cas3’s amino sequences and their genomic position, *Klebsiella pneumoniae*’s CRISPR-Cas system could be classified into type I-E and subtype I-E* [8]. Wang et al. [2] revealed that the presence of *cas1* and *cas3* in conjunction with CRISPR arrays are indicators that the isolates of *K. pneumoniae* carry the CRISPR-Cas system. It is interesting that all or some isolates with Cas3 did not have Cas1. Cas1 is typically involved in the adaptation phase of CRISPR immunity, where new spacers are integrated into the CRISPR array. The absence of Cas1 suggests potential limitations in the ability of these isolates to acquire new immunity against phages or plasmids. This phenomenon is shown in other studies such as Jwair et al. [34] and Li et al. [8] that were observed in *K. pneumoniae* or Gholizadeh et al. [35] Hullahalli [36] and Palmer [37] that were observed in *E. faecalis*. In *E. faecalis*, there are three types of CRISPR including CRISPR1-*cas*, CRISPR2, and CRISPR3-*cas*. CRISPR2 is an orphan CRISPR (lacks *cas* genes) and uses the *cas* genes of other CRISPRs. In addition, the function or activity of *cas* genes of CRISPR-Cas types of *K. pneumoniae* remains unclear and needs to be determined. In our study, 34.2% of *K. pneumoniae* isolates were found to harbor the CRISPR-Cas system which is considered a low proportion. The relatively low prevalence of CRISPR systems in this study can be attributed to the fact that most of the strains contained investigated antibiotic-resistant genes; therefore, they were found to be negative for these systems. We also found the subtype I-E* CRISPR-Cas system to have a lower prevalence (2.8%) than the subtype I-E CRISPR-Cas system (31.4%). Similar to our results, Li et al. [8] reported the prevalence of CRISPR-Cas system in *K. pneumoniae* was 30.7% (54/176) and Alcompoz et al. [38] reported 25.4% (46/181), and most of them have also belonged to type I-E. However, several studies reported the prevalence of type I-E* CRISPR-Cas system in *K. pneumoniae* was higher than in type I-E [29, 39, 40]. Furthermore, in contrast to our results, the findings of Hu et al. [39] demonstrated that the examined isolates showed co-existing type I-E and subtype I-E*. These can be because of the difference in the phylogenetic traits of bacterial isolates across different geographic areas as well as the diverse origins of the isolates used in the investigations. In this regard, Kannadasan et al. [41] reported that the type of CRISPR-Cas systems found in *K. pneumoniae* can vary greatly depending on the geographical location. Regular monitoring of the proportion of subtype I-E* and type I-E strains of *K. pneumoniae* could be crucial because it may have an impact on the global patterns of evolution and development of multidrug resistance in this bacterium [41].

Our results indicated there was a significant inverse correlation between the presence of ESBL and some AME genes in the isolates with subtype I-E CRISPR-Cas system in comparison to CRISPR-negative. The findings raised the possibility that the subtype I-E CRISPR-Cas system effectively restricts the acquisition of acquired ARGs and external DNA fragments. Similarly, Lin et al.‘s [42], Jwair et al. [34] and Wang et al. [2] demonstrated that there was a highly significant inverse association between the prevalence of CRISPR-Cas system and drug resistance in carbapenem-resistant and ESBL-producing *K. pneumoniae*. Generally, the CRISPR-Cas systems found in *K. pneumoniae* are not always correlated to a dearth of ARGs; rather, an enormous number of ARGs and CRISPR-Cas systems have been found co-existing in the analyzed genomes [38, 43]. In this regard, Alkompoz et al. [38] revealed that the frequency of the genes including *bla*_*VIM*_, *bla*_*NDM*_, *ereA2*, *armA*, *msrE*, *florR*, *mcr-3*, and *tet(B)* was significantly higher in the presence of CRISPR-Cas systems. However, other genes such as *bla*_*TEM*_, *bla*_*KPC*_, *bla*_*LAP−2*_, *rmtB*, *fosA*, and *catA3* were significantly higher in the genomes of the CRISPR/Cas-negative strains. Studies carried out on different bacterial species also have demonstrated the contradiction in the CRISPR-Cas system’s effect on preventing the spread of ARGs and, as a result, antibiotic resistance. It was previously found that the CRISPR-Cas system is significantly correlated with the absence of ARGs and high drug sensitivity in *Pseudomonas aeruginosa* [44, 45] and *Enterococcus faecalis* [35, 46]. In contrast, it was associated with increased antibiotic resistance in *Campylobacter jejuni* [47]. There are several reasons why the CRISPR-Cas system’s presence does not always prevent the spread of ARGs in bacteria. Strong selective pressure for ARGs acquisition could lead to CRISPR repression, and the presence of self-targeting spacers also may render many CRISPR-harboring strains immunologically inactive [22, 30, 32]. Moreover, phages that express anti-CRISPR proteins (Acrs) have the ability to deactivate the bacterial CRISPR-Cas system, which could lead to the spread of ARGs, as has been observed in *P. aeruginosa* [48–50]. In addition, as previously reported in *Shigella* species, insertion sequence-mediated mutations and point mutations in the *cas1* and *cas2* genes were associated with the spread of MDR strains [51]. The presence of point mutations in the protospacer adjacent motif (PAM) sequence or mismatches between invader DNA and spacer curbs CRISPR interference and drastically decreases the affinity of the cascade-crRNA complex for target DNA. This prevents the cleavage of DNA even in the presence of the CRISPR system since matched protospacer sequences are necessary for CRISPR scanning [52, 53]. For CRISPR interference activity, spacer GC content and proximity to the leader sequence are crucial because the leader sequence functions as a promotor to regulate the transcription process and is a preferred site for the insertion of further spacers [38, 53, 54]. Moreover, the restriction-modification (R-M) systems may play an important role in preventing the spread of ARGs, in addition to the CRISPR–Cas system [55]. H-NS proteins also bind to the *cas* operon’s promoter in addition to DNA-binding proteins, leading to a reduction in *cas3* expression and, as a result, a loss of CRISPR-Cas activity. Previous studies have shown that imipenem treatment induces H-NS expression, which results in a loss of CRISPR system activity [42, 54, 56–58]. Inhibition of *cas3* expression in *K. pneumoniae* through the stimulation of the transcriptional repressor H-NS leads to loss of the immunity of the CRISPR-Cas system and eventually ARGs acquisition [42]. Collectively, these reasons increase the likelihood that MGEs evade the immunity of the CRISPR–Cas system.

Within this investigation, 105 clinical *K. pneumoniae* isolates were differentiated into 100 genotypes using ERIC-PCR. This finding indicated that the great majority of isolates were not clonally related and that the spread of *K. pneumoniae* was not correlated with a clonal outbreak. The results of the ERIC-PCR technique demonstrated that isolates were highly heterogeneous and genetically diverse. This could be correlated to the genetic variation in our isolates [59, 60]. Our results were consistent with previous studies. In a study conducted in Iran, Kiaei et al. [61] differentiated the 37 *K. pneumoniae* strains into 29 genotypes using the ERIC-PCR method. In Brazil, Ferreira et al. [62] also used ERIC-PCR to differentiate 25 *K. pneumoniae* strains into 23 genotypes. In another study, Ghamari et al. [59] identified 55 and 60 different genotypes among 60 carbapenem-resistant *K. pneumoniae* isolates using RAPD and ERIC-PCR methods, respectively. We also found that the CRISPR-Cas system-containing *K. pneumoniae* isolates belonged to different clusters, and the pattern of the distribution of the ESBL and aminoglycoside genes demonstrated that there was no significant association with ERIC clusters. However, in the Wasfi et al. [60] and Kashefieh et al. [63] studies, both ERIC-PCR and RAPD-PCR genotypic analyses demonstrated an association with resistance patterns of *K. pneumonia*. Even though RAPD-PCR and ERIC-PCR are quick, easy, and affordable genotyping techniques, their reproducibility is limited and contingent upon the PCR conditions and bacterial DNA quality [63, 64]. Alternative typing techniques, like MLST, have been developed to achieve more dependable results. MLST method relies on the sequencing of conserved housekeeping genes and has demonstrated reproducibility and high reliability in comparison to other typing methods [59, 64].

## Conclusion

Our findings revealed that the presence of the subtype I-E CRISPR-Cas system is associated with the ARGs. Significantly, the isolates with subtype I-E CRISPR-Cas system had a lower frequency of the ESBL genes and some AME genes compared to CRISPR-negative isolates. Analysis of the correlation between the CRISPR-Cas system and antibiotic resistance will help to identify and better understand the mechanism of bacterial resistance and provide new instructions for the prevention and treatment of bacterial antibiotic resistance. Therefore, the CRISPR-Cas system along with other genetic markers could be used for infection control by resistant pathogens, to give insights into their genetic contents and phenotypic characteristics, and also to differentiate low-risk strains of pathogens from high-risk strains.

## Data Availability

All data generated or analyzed during this study are included in this published article.
